# Progress in quality control, detection techniques, speciation and risk assessment of heavy metals in marine traditional Chinese medicine

**DOI:** 10.1186/s13020-023-00776-y

**Published:** 2023-06-16

**Authors:** Yuan-sheng Guo, Tian-tian Zuo, An-zhen Chen, Zhao Wang, Hong-yu Jin, Feng Wei, Ping Li, Shuang-cheng Ma

**Affiliations:** 1 National Institutes for Food and Drug Control, No. 31 Huatuo Road, Daxing District, Beijing, 102629 China; 2 China Pharmaceutical University, Nanjing, 211198 China; 3 NMPA Key Laboratory for Quality Research and Evaluation of Traditional Marine Chinese Medicine, Qingdao Institute for Food and Drug Control, Qingdao, 266073 China

**Keywords:** Marine traditional Chinese medicines (MTCMs), Heavy metals, Risk assessment, Detection techniques, Removal techniques, Quality control

## Abstract

Marine traditional Chinese medicines (MTCMs) hold a significant place in the rich cultural heritage in China. It plays an irreplaceable role in addressing human diseases and serves as a crucial pillar for the development of China's marine economy. However, the rapid pace of industrialization has raised concerns about the safety of MTCM, particularly in relation to heavy metal pollution. Heavy metal pollution poses a significant threat to the development of MTCM and human health, necessitating the need for detection analysis and risk assessment of heavy metals in MTCM. In this paper, the current research status, pollution situation, detection and analysis technology, removal technology and risk assessment of heavy metals in MTCM are discussed, and the establishment of a pollution detection database and a comprehensive quality and safety supervision system for MTCM is proposed. These measures aim to enhance understanding of heavy metals and harmful elements in MTCM. It is expected to provide a valuable reference for the control of heavy metals and harmful elements in MTCM, as well as the sustainable development and application of MTCM.

## Highlights

- An overview of the research on heavy metals and hazardous elements in marine traditional Chinese medicine (MTCMs) was analyzed.
- The current status of heavy metal pollution in marine traditional Chinese medicine (MTCMs) and its hazards were studied.
- The corresponding morphological and valence studies of heavy metals and hazardous elements in marine traditional Chinese medicine (MTCMs) and marine organisms were analyzed.
- The risk assessment of heavy metals and harmful elements in marine traditional Chinese medicine (MTCMs) and marine organisms was conducted.
- A pollution detection database and quality and safety supervision system for marine traditional Chinese medicine (MTCMs) were analyzed and established.

## Introduction

The ocean, covering two thirds of the Earth's surface, is a natural treasure trove for humanity, boasting abundant biological resources. Marine organisms make up approximately 80% of the total global species [1], with a biomass accounting for around 87% of the world's total biomass [2]. Due to their unique ecological environment, characterized by high pressure, high salt, low temperature and limited light [3], marine organisms possess distinctive biological and pharmaceutical advantages when compared to terrestrial organisms. It provides a vast repository of drug compounds that can be utilized worldwide to combat tumors, immune system diseases, cardiovascular diseases, and neurological diseases, indicating future research directions [4]. MTCM is an important component of traditional Chinese medicine culture and is one of the main driving forces for the advancement of traditional Chinese medicine (TCM) in China. MTCM refers to medicinal substances derived from the ocean (including the ocean itself, as well as coastal beaches, wetlands, and saline areas influenced by the ocean) that are used for the prevention and treatment of diseases under the guidance of TCM theory. China is the country that first developed and utilized MTCM in the world. As early as the Spring and Autumn Period and Warring States Period, Chinese literature such as "Zhou Li", "Shi Jing", and "Shan Hai Jing" recorded information about MTCMs. In recent years, with the development of the TCM industry, the issue of safety has been increasingly emphasized, particularly the heavy metal and harmful element residues in TCM, which has become a social concern internationally. However, there has been limited research on heavy metals and harmful elements in MTCM, therefore scholars still need to conduct more in-depth research in this direction.

China is a vast maritime nation boasting an impressive 18,000-kilometer coastline and 3 million square kilometers of sea area, with abundant marine resources. It provides unique conditions for the development of MTCMs in China, making it a key element of China's development strategy. Therefore, accelerating the construction of a "blue pharmacy" and vigorously developing the MTCM industry has become a top priority for China's economic development. However, in recent years, the rapid development of the social economy has resulted in increasingly severe marine pollution, particularly heavy metal pollution, which has had a serious impact on the marine ecological environment. Heavy metals not only contaminate the marine environment, but also have detrimental effects on marine organisms. After heavy metals residues are ingested by marine organisms (including marine animals and marine plants) in the ocean, it is difficult to degrade the heavy metals themselves, leading to a range of issues, such as the accumulation of excessive heavy metals and severe pollution in marine organisms and MTCMs. As a result, humans can be seriously harmed through the transmission of heavy metals up the food chain. The 2020 edition of the Chinese Pharmacopoeia has established a corresponding detection system for heavy metals and harmful elements in terrestrial Chinese medicines. However, the quality standards for MTCMs in China remain inadequate, and only 11 MTCMs are included in the 2020 edition, which does not reflect the abundant marine resources in China. Furthermore, the number of MTCMs included in the contemporary marine publication “Chinese Marine Materia Medica” differs significantly, and the quality standards of MTCMs included in the 2020 edition of the Chinese Pharmacopoeia have serious missing entries. In addition, from the existing reports on MTCMs, there are fewer studies on the pharmacological basis of MTCMs, which hinders their clinical application to some extent.

**Fig. 1:**
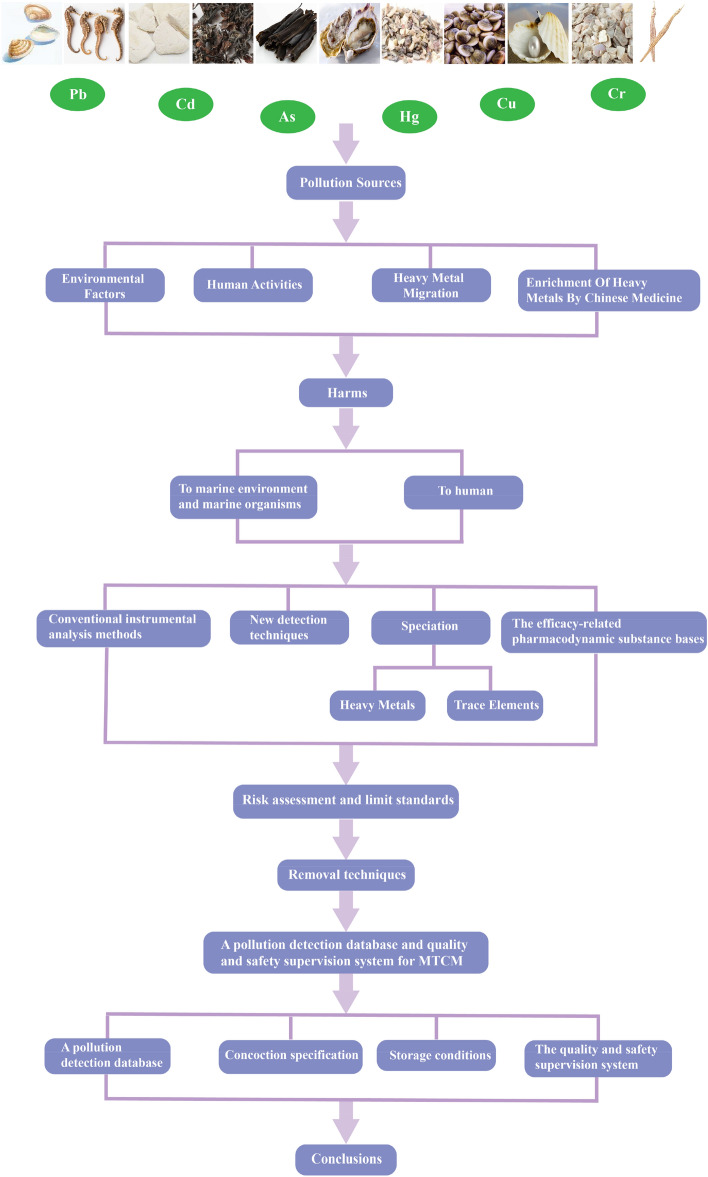
Schematic diagram of the interactions between heavy metals and MTCMs

This paper firstly analyzed the current situation of research on MTCMs in China, discussed the sources of pollution, pollution levels, and hazards to marine organisms and humans caused by heavy metals and harmful elements in MTCMs, analyzed the detection and analysis techniques of heavy metals and harmful elements in MTCM and marine environment, and conducted risk assessment of heavy metals and harmful elements in MTCM, further clarified the limit standards of heavy metals and harmful elements in MTCM, summarized the control techniques of heavy metals and harmful elements in MTCMs, described the progress of quality control in MTCMs, and proposed the establishment of a pollution detection database and a comprehensive quality and safety supervision system for MTCMs. The aim of this paper is to provide references for the control of heavy metals and hazardous elements in MTCMs, as well as for the sustainable development and application of MTCMs (as shown in Fig. 1).

## Current status of research on MTCMs in China

**Fig. 2:**
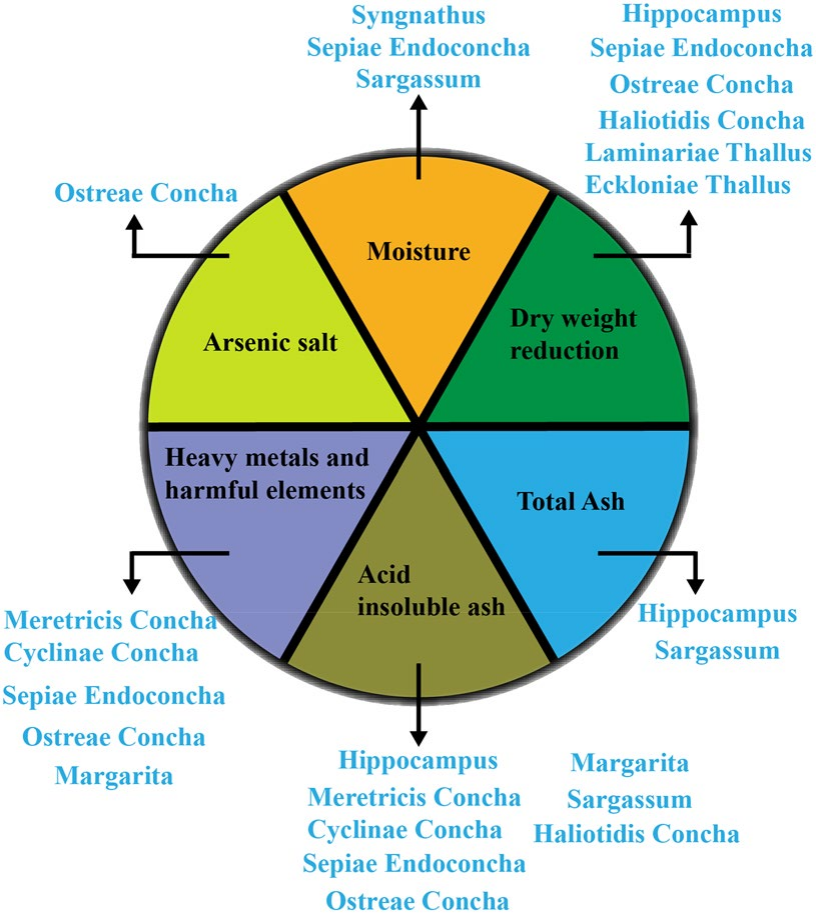
MTCMs inspection items in the 2020 edition of the Chinese Pharmacopoeia and relevant standards for Chinese herbal medicines

China has a rich history of researching MTCMs, and the biological activity of MTCM is remarkable. Recent studies on MTCMs have shown that the current research on MTCMs in China is increasing, but there are still many shortcomings. Firstly, the quality standards for common MTCMs in China remain imperfect. For instance, while the marine writings “Chinese Marine Materia Medica” contains 613 MTCMs, the 2020 edition of the Chinese Pharmacopoeia only includes 11 MTCMs, such as syngnathus, hippocampus, meretricis concha cyclinae concha, sepiae endoconcha, ostreae concha, haliotidis concha, arcae concha, margaritifera concha, sargassum, laminariae thallus eckloniae thallus, and margarita, which is seriously incompatible with the abundant marine resources in China, and the regional Chinese medicine standard includes only 60 MTCMs, meaning that about 90% of MTCMs in China do not have standardized quality standards. Furthermore, the quality standards of MTCMs included in the 2020 edition of the Chinese Pharmacopoeia still have serious missing entries (as shown in Fig. 2). Secondly, the bioactive components of MTCMs in China need to be explored in-depth. Marine drugs and MTCMs have certain irreplaceable qualities in active development compared to terrestrial Chinese medicines due to the characteristics of high pressure, high salt and low light in marine environment. Some studies have found that MTCMs have pharmacological effects such as anti-viral, anti-tumor, anti-inflammatory, and treatment of cardiovascular and neurological diseases [5]. However, the research on the activity of MTCMs in China is still insufficient, to a certain extent, it limits the clinical application of MTCMs. Additionally, with the rapid development of social economy, Ocean pollution has become increasingly severe, leading to the inevitable contamination of MTCMs by heavy metals and harmful elements in the marine environment. Although China has established a corresponding detection system for heavy metals and harmful elements in terrestrial Chinese medicines, the detection of heavy metals and harmful elements for MTCMs is still incomplete, especially in terms of elemental analysis and speciation of MTCMs, which is currently scarce. Therefore, from a comprehensive perspective, the research on MTCMs in China is still in its early stages, and there is significant potential for further research by scholars.

## Pollution status and harm of heavy metals in MTCMs

### Pollution sources and levels of heavy metals and harmful elements in MTCMs

**Fig. 3:**
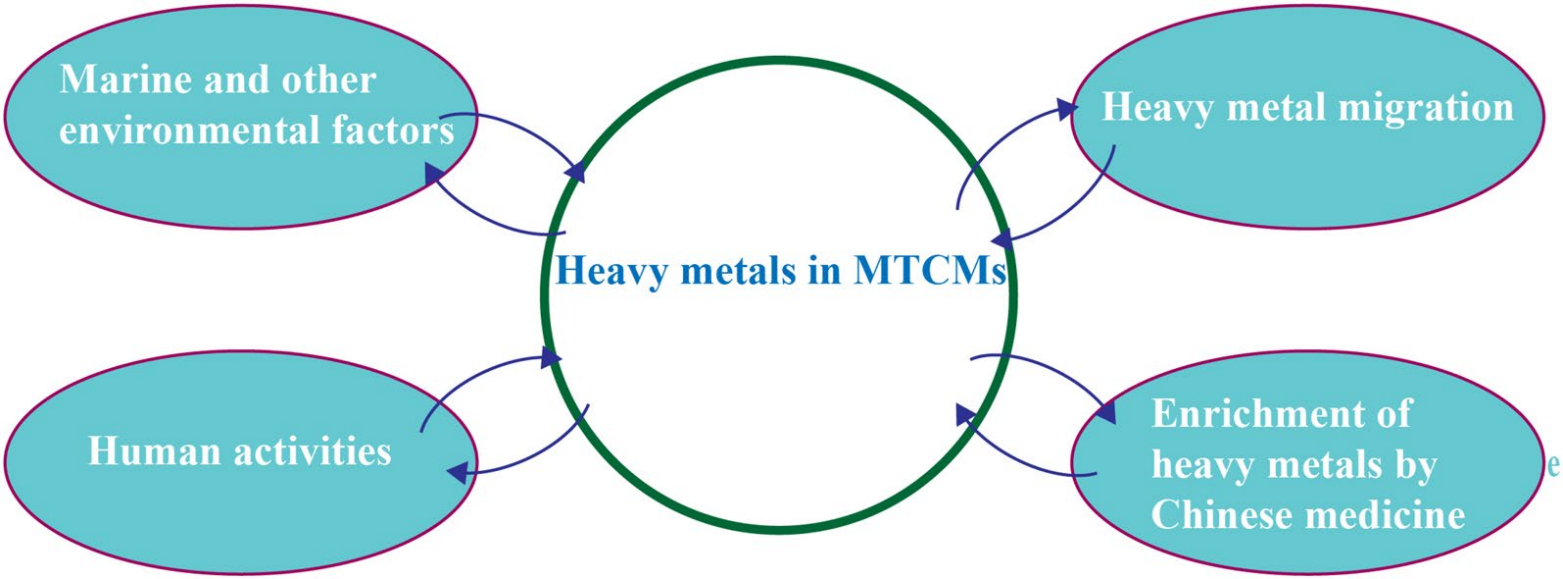
Sources of heavy metal pollution in MTCMs

Heavy metals generally refer to chemical elements with a density over 5.0 g/cm^3^. Common heavy metals mainly include lead (Pb), chromium (Cr), arsenic (As), mercury (Hg), zinc (Zn), copper (Cu), cadmium (Cd), and others. Different heavy metals have varying pollution levels in different sea areas. The survey revealed that the average concentration of As in seawater ranged from 1.00 to 2.00 ug/L, Hg concentration ranged from 0.02 to 3.61 ug/L, Pb concentration ranged from 0.10 to 1.49 ug/L, and Cr concentration ranged from 0.10 to 4.16 ug/L [6]. For instance, Tian K et al. [7] conducted an ecological risk assessment of heavy metals in coastal sediments and water of the Bohai Sea and Yellow Sea. The geological cumulative index (I_geo_) and pollution load index (PLI) indicated that the Bohai Sea sediments displayed high Hg and Pb pollution, the Yellow Sea of China exhibited high As, Hg and Pb pollution, and the Yellow Sea of Korea displayed high Cd and Hg pollution. In recent years, with the development of industrialization, the ocean has been increasingly polluted. Heavy metals and harmful elements pose a significant threat to the marine environment and organisms. Once marine organisms have absorbed heavy metals, these substances can become magnified as they move up the food chain, ultimately posing a potential hazard to human health [8]. Thus, the research on detecting and controlling heavy metals and harmful elements in MTCMs is becoming increasingly urgent. Several factors contribute to the pollution of heavy metals and harmful elements in MTCMs, including natural and human factors (as shown in Fig. 3), the pollution sources are described from the following four aspects.

#### Pollution caused by marine and other environmental factors

MTCM is significantly impacted by the ocean since it grows in the ocean or in areas such as seaside beaches and wetlands that are influenced by the ocean for extended periods. Studies have found that the pollution level of seawater and seabed sediments can directly reflect the pollution level of marine organisms and MTCM [9], and heavy metals can enter the marine ecosystem through natural factors, such as volcanic eruptions, crustal weathering, atmospheric deposition, and other ways [10], causing pollution to seawater and MTCM, especially to near-shore marine ecosystems. Furthermore, heavy metals may also cause pollution in sea areas far from the shore through the transport of large aerosols and suspended particles [11]. The concentration of As shows less variation in offshore areas compared to nearshore areas, where it tends to increase due to factors such as river inputs, changes in salinity, and pH. For example, the average concentration of As in seawater ranges from 1 to 2 ug/L, while the arsenic content in the Rhone estuary in France ranges from 1.3 to 3.7 ug/L [12]. In 2002, the As concentrations in the Yellow Sea and East China Sea were influenced by the input of freshwater from the Yangtze River, resulting in concentrations of 1.12 ug/L and 1.21 ug/L, respectively [13]. Furthermore, heavy metal concentrations in marine sediments are relatively higher and can serve as potential sources of seawater pollution. The average concentrations of As and Cr in marine sediments are around 40 ug/L [14]. For example, As concentrations in the East China Sea range from 1.70 to 22.1 ug/L [15], while in Baltimore Harbor in the United States, As concentrations range from 25.0 to 41.1 ug/L [16]. Cr concentrations in samples collected in Shantou, China, ranged from 36.1 to 74.2 ug/L [17].

#### Pollution caused by human activities

In recent years, the rapid development of industrialization and urbanization has led to anincrease in the mining and processing of heavy metals, industrial emissions, coal combustion, automobile exhaust emissions, as well as marine transportation, marine oilfield mining [18] and other human activities. This has resulted in varying degrees of pollution in the marine environment, which pose a threat to humans at the top of the food chain [19].

#### Pollution due to heavy metal migration

Heavy metals in the ocean accumulate in the human body through the food chain (water body—marine plants—marine animals—human body) [20], which increases the risk of heavy metals and harmful elements. Heavy metals can continuously migrate and transform in the water through mediums such as seawater, biological media, and seafloor sediment, as well as through processes such as precipitation, adsorption, and complexation. They exhibit biogeochemical behavior, for example, Hg can migrate and transform in three media: atmosphere, soil and seawater, and ultimately harms human health through the accumulation in organisms and the transfer of the food chain. Different heavy metals may have different migration pathways. For example, Wu Hao et al. [21] used ICP-MS to detect and analyze the heavy metal content of 13 marine organisms in Xiamen Bay waters, and found that the detection rate of Pb in seafloor sediments was higher than that of As, while the detection rate of As in marine organisms was higher than that of Pb in marine organisms. In addition, the migration and residues of heavy metals in marine organisms are related to the seasons. By comparing the heavy metal residual amounts in fish in different seasons, it was found that the residues of Cr, Ni, Cu and Pb were higher in summer than in winter, while the residues of Zn were higher in winter than in summer.

#### Contamination caused by the species-specific enrichment of Chinese medicine for heavy metal elements

MTCMs mainly consist of marine animals, marine plants and marine minerals, which are often rich in heavy metals due to the species specificity of TCM. Marine plants and marine animals are easy to accumulate heavy metals in specific parts due to individual differences and varying degrees of metabolism in different tissues and organs, resulting in pollution. Different types of MTCMs often have varying abilities to enrich heavy metals [22]. For example, ARISEKAR et al. [23] determined the contents of Pb, Hg, Cd, Cr, Cu, Zn and other heavy metals in seven types of macroalgae in Mannar Bay in southern India. They found that the brown algae had a higher capacity to enrich these heavy metals than red algae and green algae, but the relative contents of Pb and Hg in red and green algae were higher. Liu B et al. [24] investigated the production, trophic magnification factor and health risk of seven heavy metals in 20 marine organisms in Laizhou Bay, China. They found that the total concentration of seven heavy metals in organisms followed this order: crab > shellfish > algae > fish > starfish. Cd was the most bioaccumulative element, and the heavy metal accumulation ability of crabs and shellfish was much higher than that of algae, fish and starfish.

### Harm of heavy metals and harmful elements in MTCMs

#### Harm of heavy metals and harmful elements in MTCMs to marine environment and marine organisms

Heavy metals and harmful elements enter the marine ecosystem and are the primary pollutants in the ocean, resulting in significant impacts on the marine environment, marine organisms and human health. Even trace concentration of heavy metals in natural marine waters can be toxic, such as Hg and Cd, which can cause toxicity in the concentration range of 0.01–0.001 mg/L. Due to their accumulative, persistent, and long-term nature, heavy metals pose a potential risk and can synergistically interact with other pollutants to contribute to the decline of marine biological resources. For example, the increase of heavy metals and harmful elements can affect the gender and growth of fish in the ocean, posing a threat to their survival. Marine plants, such as sargassum, can accumulate heavy metals to varying degrees in aquatic systems. As primary producers at the bottom of the food chain, they are consumed by various organisms and may cause toxic effects. For instance, Baumann indicated that heavy metals, such as Cu, Cd and Zn can inhibit chlorophyll photosynthesis and growth in sargassum by affecting photosystem complexes, cytochrome complexes, and plastocyanin [25].Furthermore, scientific literature has reported that heavy metals and harmful elements can result in abnormal development of marine organisms, low survival rates of larvae, and DNA mutations in organisms, ultimately affecting marine biodiversity and the integrity of the food chain.[26]

#### Harm of heavy metals and harmful elements in MTCMs to human

Heavy metals and harmful elements in MTCM pose a potential threat to human beings at the top of the food chain due to their transmission and amplification through the food chain. Numerous studies have shown that excessive intake of Cu can lead to anemia, coronary heart disease, female infertility and other diseases, as well as cause damage to human skin, stomach and respiratory tract [27]. China is one of the countries facing severe Hg pollution, with organic and inorganic forms of Hg being the predominant types found in the food chain. Inorganic mercury can be converted into methylmercury, which has the highest toxicity and accumulates easily in organisms through biological or abiotic action. Methylmercury is fat-soluble and can penetrate the blood–brain barrier [28], leading to significant deposition in brain area [29] and cell damage in specific area. It can also pass through placental barrier and affect fetal development [30]. Low concentrations of methylmercury can cause cardiovascular and cerebrovascular diseases, while long-term exposure can impact the pituitary and liver, leading to immune system diseases [31]. For instance, the Minamata disease in Japan resulted in serious poisoning from consuming seafood contaminated with methylmercury, affecting hundreds of thousands of people. Cd is a carcinogen with high toxicity. Excessive exposure to Cd can result in kidney, liver and respiratory tract injuries, and in severe cases, may lead to cardiovascular disease, nervous system disease and osteoporosis [32]. Studies have shown that As exists in two main forms inorganic As and organic As, with inorganic arsenic being more toxic and easily absorbed by the human body, while organic As is excreted faster and does not accumulate easily. Excessive intake of inorganic As may cause skin injuries, cardiovascular disease, atherosclerosis, and other diseases [33]. The toxicity of Pb is dependent on its chemical form, with organic Pb being more toxic than inorganic Pb. Excessive ingestion of Pb by the human body can result in anemia, kidney damage, and nervous dysfunction. Moreover, lead may also affect the nervous system of fetuses, leading congenital mental retardation. Cr exists in different forms, such as Cr^3+^ and Cr^6+^ [34]. Some studies suggest that when Cr content exceeds safety limits, it may have carcinogenic effects, with Cr^6+^ being a natural carcinogen with the highest toxicity. Under alkaline conditions, Cr (III) may be oxidized into Cr (VI) [35]. Shaheen et al. [36] discovered that Cr (VI) usually exists in the form of chromate oxyanion ([CrO4]^2−^), which interacts with ascorbate and sulfhydryl upon entering cells, leading to the release of toxic by-products, irreversible DNA damage, malignant cell transformation [37], and further harm to the human body.

## Research progress in the detection and analysis technology of heavy metals and harmful elements in MTCMs and marine organisms

The pollution of marine environment and MTCMs by heavy metals and harmful elements is a growing concern, posing a significant threat to human health. Detecting heavy metal elements and understanding MTCM pollution accurately and swiftly is crucial. Preventing and controlling heavy metal pollution and improving the clinical application of MTCMs hold great significance. In recent years, scientific researchers have made notable contributions to detection technology in China by developing new methods that combine characteristics from different disciplines. These methods aim to achieve more convenient, rapid, and accurate determination of heavy metals, hazardous elements, and trace elements in MTCMs to meet the increasing demand for efficient detection in the field.

### Conventional instrumental analysis methods in MTCMs and marine organisms

Currently traditional analytical methods for detecting heavy metals include inductively coupled plasma mass spectrometry (ICP-MS), inductively coupled plasma atomic emission spectrometry (ICP-AES), atomic fluorescence spectrometry (AFS), atomic absorption spectrometry (AAS), X-ray fluorescence spectrometry (XFS), infrared spectrometry, ultraviolet–visible spectrometry, and ion chromatography, etc., which are shown in Table 1.

**Table 1:** Comparative summary of major heavy metal detection techniques in MTCMs

Detection method	Limit of detection	Detection sensitivity	Analysis elements at the same time	Time of analysis	Advantages	Disadvantages	References
ICP-MS	10^–15^-10^–12^	Higest	Multiple	All elements 2–6 min/sample	Fast analysis speed. With lower detection limits Wide range of detection High degree of automation Wide dynamic linear range	High cost Samples generally need to be pre-transformed into solution Some light elements (e.g.,S, Ca, Fe, K, Se) have serious interference in ICPMS	[82] [83] [84] [85] [86]
ICP-AES	10^–9^	Low	Multiple	5–30 elements/minute/sample	High sensitivity Low limit of detection Wide range of detection	Higher equipment and operating costs Large argon consumption of working gas	[87] [88]
AFS	10^–9^	High	Multiple	6–10 min/sample	High sensitivity Low limit of detection Fewer interferences Wide dynamic linear range Simple score lines	Smaller detection rnge	[89]
AAS	10^–12^–10^–9^	High	Single	3–4 min/sample	High selectivity High sensitivity Wide range of detection High anti-interference ability	Unable to achieve simultaneous quantitative analysis of multiple elements Narrow linear range Expensive instruments	[90] [91]
XRF	10^–9^	Low	Multiple	5–20 s/sample	Simple operation Rapid detection	Weaker technology	[92] [88]

### New detection techniques and methods in MTCMs and marine organism

While traditional detection techniques such as ICP-MS, ICP-AES, AFS, AAS, XFS, infrared spectrometry, ultraviolet–visible spectrometry, and ion chromatography offer accurate results for heavy metals and harmful elements content, they do have limitations. These methods require complex sample pre-treatment, expensive equipment, longer detection times, and confined to laboratory settings. As a result, researchers are continuously exploring and developing new techniques for rapid heavy metal detection. Emerging techniques include electrochemical analysis methods such as dissolution voltammetry, potentiometric dissolution, polarimetric analysis, and electrochemical sensor method. Additionally, biological detection methods such as immunoassay, enzyme analysis, biosensor methods, and microbial detection methods are also being investigated for heavy metal detection. These novel approaches aim to overcome the limitations of traditional methods and enable faster and more convenient heavy metal detection.

### Study on the speciation of heavy metals and harmful elements in MTCMs and marine organisms

In the study of metal and heavy metal elements in the ocean, it has been observed that not all metal elements are harmful to marine organisms and humans, so it is necessary to classify common metal elements into two categories based on their health effects on humans: trace elements and toxic elements. Trace elements consist of both essential and non-essential elements for the human body, such as iron (Fe), Cu, Zn, selenium (Se), and others. These elements play important roles in human metabolism, and their effects are dependent on their concentrations in the body. On the other hand, toxic elements include As, Pb, Hg, Cd, Cr, and others. These elements can cause varying degrees of harm to human health, and the extent of harm is not only determined by the concentration of these elements, but also by their speciation or chemical form. Proper classification of metal elements into trace elements and toxic elements is essential in understanding their impact on marine organisms and human health, and in devising appropriate strategies for monitoring and managing metal pollution in marine environments.

#### Study on arsenic speciation

In recent years, research on heavy metals has increasingly focused on speciation studies. This article describes various studies on As, both domestically and abroad. For instance, Ferrante et al. [38] examined fresh seafood from the Mediterranean and European Atlantic coasts and observed that arsenical concentrations were highest in mollusks, followed by pelagic animals and benthic animals. Similarly, Filippini et al. [39] and Traina et al. [40] discovered that shellfish and cephalopod marine animals have higher concentrations of As compared to other marine organisms. Liobet et al. [41] found that while marine organisms may contain high levels of As, the majority of the detected As is typically in the form of non-toxic total As, rather than inorganic As. Meharg et al. [42] found that inorganic As in fish was negligible, with only a few species reaching concentrations of 1–5%. Mania et al. [43] similarly found that the amount of inorganic As in fish is very low, suggesting that consuming fish does not pose a significant health risk to humans. Kalia et al. [44] identified four oxidation states of As in the marine environment, As^5+^, As^3+^, As^0^, and As^3−^. As (III) and As (V) are the most important oxidation states, while As^3−^ is only found in the environment with low potential value. Zhang W et al. [45] reported that As exists in two oxidation states: arsenite [As_2_O_3_. As(III)] and arsenate[As_2_O_5_. As(V)], which can be converted between each other through redox and methylation reactions. As(III) is approximately 60 times more toxic than As(V), whereas As(V) is about 70 times more toxic than methylated substances such as monomethylarsenic acid (MMA) and dimethylarsenic acid (DMA). Additionally, their study investigated the bioaccumulation and subcellular distribution of As in the muscle tissue of *Terapon jarbua*, revealing that AsB accounted for 89–97% of the total As content, while DMA, As (III), and As (V) were present at lower concentrations of approximately 1.0–2.3%, 1.1–8.6% and 0.9–3.4%, respectively. This suggests that *T.jarbua* can convert inorganic As into organic As in vivo. Moreover, the predominant arsenic species found in total As in *T.jarbua* was organic As, mainly arsenic betaine (AsB), followed by DMA, As (III), As (V) and MMA. The concentrations of methylated and inorganic As were very low. Amlund et al. [46] also observed that arsenobetaine (AsB) was the primary arsenic species in Atlantic salmon and Atlantic cod, accounting for more than 99% of the total As. Similarly, Francesconi et al. [47] found that AsB, which has low toxicity, constituted more than 95% of all As compounds in marine fish.

#### Study on mercury speciation

Hg is a toxic element that can be detected in almost all marine organisms, as shown by Cheng et al. Additionally, Liu et al. [48] demonstrated that Hg is present in the marine environment in both organic Hg (OHg) and inorganic Hg (Hg^2+^) forms [49], with methylmercury being the most dominant. Yaghmaeian et al. [50] revealed that certain bacteria can convert inorganic Hg to toxic methylmercury, which can have serious adverse effects on the human nervous system and developmental processes [51]. While methylmercury is more likely to accumulate in fish and humans, inorganic Hg is not toxic to humans due to its rapid excretion and low concentrations [52]. Cooking can reduce mercury, with Hg^2+^ being reduced to a greater extent than methylmercury, as demonstrated by Wen Liao et al. [53]. Furthermore, the degree of bioaccumulation of mercury is positively correlated with the size and age of the fish [54]. In contrast, other elements, such as As, Pb and Cd, have an opposite correspondence between the degree of bioaccumulation and the size of the organism [55].

#### Study on speciation of other elements

Olaifa et al. [56] demonstrated that high concentrations of copper can have toxic effects on marine organisms due to the formation of complexes between Cu^2+^ and organic and inorganic substances, resulting in toxicity.

Chandra et al. [34] studied the various oxidation states of Cr in nature, ranging from Cr^2+^ to Cr^6+^, with Cr(III) easily oxidizing to Cr(VI) at high oxygen concentrations. Cr(VI) is highly toxic, more soluble in water than other forms, and can easily cross cell membranes.

Pb can exist in the marine environment as organic or inorganic Pb [57], with organic Pb being more toxic than inorganic Pb. Most Pb in the marine environment is organic, accounting for about 50%-70% of the total Pb. Some studies have reported that the more methyl or ethyl groups attached to the Pb molecule, the greater the toxicity produced [58].

### Study on the speciation of trace elements in MTCMs and marine organisms

In MTCMs, especially those derived from animals, there are various trace elements, such as Se and Zn, that have been reported to be important substances for the pharmacological activities of MTCMs, including anti-cancer and anti-tumor effects. Zhang Yan et al. [59] utilized the HPLC-HG-AFS method to investigate the selenium morphology in anti-cancer MTCMs, such as ostreae concha, meretricis concha cyclinae concha, green clam, and sea cucumber. The results revealed that these MTCMs are rich in Se, predominantly containing beneficial organic forms of selenium, such as Se(IV), MeSeCys, SeCys2, and SeMet.

### Research on quality control of speciation of heavy metals and harmful elements in MTCMs

Research has found that the toxicity characteristics of heavy metals are closely related to their speciation. The speciation of heavy metals has gradually become a new research hotspot in this field. Therefore, the detection and analysis of speciation of heavy metals and harmful elements in MTCMs are extremely important for their quality control. Chen Lingxin's team proposed a novel colorimetric nanosensor strategy for Hg speciation based on the aggregation of gold nanoparticles (Au NPs) induced by analyte in the presence of dithiocarbamate (DDTC) ligand with thiol group [60]. The results showed that this strategy is simple, rapid, and has specific selectivity towards Hg species, allowing effective differentiation between organic and inorganic Hg, with higher detection capability compared to conventional methods. Liu et al. [61]designed a fluorescent reaction using DNA-gold nanoparticles and OliGreen to detect Hg^2+^, demonstrating high selectivity and suitability for water sample analysis. Research has shown that HPLC combined with ICP-MS for chromium speciation analysis can achieve very low detection limits (0.3–0.5 μg/L) while effectively analyzing Cr speciation. Powell et al. achieved separation of Cr(III) and Cr(VI) by combining high-pressure liquid anion chromatography (with nitric acid as eluent) with direct injection nebulization and ICP-MS, with detection limits of 30 and 60 ng/L for Cr(III) and Cr(VI), respectively. Hossain et al. [62] developed a method for simultaneous determination of Cr(III) and Cr(VI) based on the principle of formation of different complexes with pyrrolidine dithiocarbamate ammonium (APDC). The results showed excellent reproducibility of this method. Luca Guerrini's team achieved qualitative and quantitative analysis of inorganic mercury (Hg^2+^) and methylmercury (CH_3_Hg^+^) using SERS sensor for the first time [63]. Currently, there is limited research on the speciation of heavy metals in MTCMs, making it a potential future research direction and an important branch of MTCMs research.

## Risk assessment of heavy metals and harmful elements in MTCMs and marine organisms

Heavy metals and hazardous elements pose a severe threat to human health, and conducting scientific assessments of their health risks in MTCMs and marine organisms is crucial for regulatory decisions by governmental departments. Zuo Tian-tian et al. [64] used ICP-MS to determine the contents of Pb, Cd, As and Hg in TCMs. The first risk assessment strategy was employed to evaluate the risk of TCMs by calculating the estimated daily intake (EDI), target hazard quotient (THQ), and cancer risk (CR).The results from THQ values indicated that most TCMs did not pose any health risk to humans, except for Pheretima and Turtle A which showed potential health risks. The CR results also indicated that the carcinogenic risk from Pb and As in animal herbal medicines, except Pheretima, was below the acceptable lifetime risk. In 2021, Zuo Tian-tian et al. [65] developed a refined risk assessment strategy for heavy metals in TAMs based on bioavailability. The strategy incorporated factors such as bioavailability level of heavy metals, exposure time of TAMs, storm rate frequency, and safety factors, resulting in a more realistic and accurate assessment of the health risk of Cd in TAMs. This new approach provides valuable reference for assessing the health risk of heavy metals and harmful elements in MTCMs. Bakhshalizadeh et al. [66] demonstrated that As poses a carcinogenic risk to children that exceeds the "acceptable" threshold. This conclusion was drawn from their analysis of heavy metals in the muscles of Chelon auratus and Chelon saliens, as well as their evaluation of potential risks to human health. Storelli MM et al. [67] conducted a study in Italy, analyzing the levels of heavy metals in five common bottom fishes and assessed the risk using the target hazard coefficient method (THQs). While the non-carcinogenic risk analysis indicated that all fishes were safe for human consumption (THQ < 1), the study found that Hg levels exceeded the recommended limit of 1, and the accumulation of Hg was correlated with fish size, suggesting potential health risks for human consumption. Khandaker et al. [68] analyzed the content of heavy metals in basic food seaweed in East Asian countries and evaluated the potential non-carcinogenic and carcinogenic risks. The results revealed that the non-carcinogenic risks associated with most heavy metals (such as Pb, Cr, As, etc.) exceeded the unified value of hazard coefficient, indicating that long-term consumption of seaweed may have adverse effects on health. Additionally, the carcinogenic risk value of Central Africa elements exceeded the reference limit set of US-EPA. Chen Bin et al. [69] investigated the pollution of seven heavy metals (Hg, As, Cu, Pb, etc.) in the surface sediments of the outer shelf waters of the Pearl River Estuary in November 2017. They used the Hakanson potential ecological risk index method to quantitatively assess the potential ecological risk of heavy metals in sediments, and the results revealed that the sediment environment in the study area had a low potential ecological risk, with the risk coefficient of Cd in the western surface sediments reaching a medium potential ecological risk. Kumar et al. [70] conducted an ecological risk assessment of heavy metals in seawater from the southeast coast of India. Their findings showed that the Cu content in most of the samples exceeded the acute maximum and chronic continuous concentration standards set by the U.S. EPA, while other heavy metal contents met the criteria for Class I seawater. Most of the ERI values (ranging from 0.46 to 3.99) in this sea area were classified as ecologically low risk level for heavy metals.

## Limits of heavy metals and harmful elements in marine medicine and MTCM

Given that heavy metals and harmful elements present in MTCMs and marine organisms pose a serious threat to human health, it is necessary to strictly regulate their levels in order to protect human life and health. In some areas, the content of heavy metals in MTCMs exceeds the set limit, which is why domestic and foreign authorities have established relevant standards for the content of heavy metals in marine organisms, as detailed in Table 2. While there is no unified international standard for limits on heavy metals and harmful elements in marine organisms due to the vast number of species, different limits are set based on their specific characteristics and research findings. As heavy metal detection technology, risk assessment, and MTCMs continue to develop rapidly, there is a need to gradually establish international standards for heavy metals and harmful elements in marine organisms and MTCMs to enhance supervision and management. Furthermore, the Chinese Academy of Traditional Chinese Medicine Resource Center established the first ISO standard for heavy metals in herbal medicines, titled “ISO 18664: 2015 Traditional Chinese Medicine—Determination of heavy metals in herbal medicines used in Traditional Chinese Medicine” (TCM—Limits of heavy metals in herbal medicines), which was officially implemented in 2015 [71]. Guo Lanping et al. [72] utilized the ISO international standard as the basis for "Chinese medicine—Heavy metal limit for Chinese herbal medicines", and found that animal herbs had the highest exceedance rates for Pb, As, Cd, and Hg, particularly in marine animal medicines. For instance, Feng Jiang et al. [73] discovered that in Shaanxi, Gansu, and other regions oyster, stone cassia, turtle plate, and squid bone had Pb contents of 18.18 mg/kg, 14.35 mg/kg, 13.68 mg/kg, and 10.47 mg/kg, respectively. Regarding As, the exceedance rate was 16.67%, with the highest As content (17.87 mg/kg) being found in the ground dragon of unknown origin, as well as in two batches of leeches (8.81, 5.80 mg/kg), geodon of unknown origin (4.55 mg/kg)[74], and sargassum (81.34–82.55 mg/kg)[75]. For Cd, the exceedance rate was 28%, with the Cd content in ostreae concha, sepiae endoconcha, haliotidis concha, turtle plate, and geodon being 4.52 mg/kg, 4.13 mg/kg, 3.94 mg/kg, 2.55 mg/kg, and 2.64–3.04 mg/kg, respectively [76].

**Table 2:** Domestic and international standards for heavy metals and harmful elements in marine organisms

Country (Institution)	name of the standard	Lead (Pb)	Cadmium(Cd)	Methylmercury (Hg)	Arsenic (As)	Chromium (Cr)
National Codex Alimentarius Commission (CAC)	General Standard for Contaminants and Toxins in Food and Feed	Fish: 0.3	Bivalve mollusks: 2 Cephalopoda: 2	Tuna: 1.2 Alfonsino: 1.5 Marlin: 1.7 Shark: 1.6		
European Union(EC)	Commission Regulation (EC) No 1881/2006	Fish: 0.3 Crustaceans: 0.5 Bivalve mollusks: 1.5 Cephalopoda: 1.0	Fish: 0.05 Carnivorous fish: 0.1 sailfish: 0.3 crustaceans: 0.5 Bivalve mollusks: 1.0 Cephalopoda: 1.0	Aquatic products and fish: 0.5 carnivorous fish: 1.0		
China	National food safety standard-Limits for contaminants in food (GB2762—2022)	fresh algae: 0.5 Algae products: 1.0 Fish: 0.5 bivalve: 1.5	Fish: 0.1 crustaceans: 0.5 Bivalves、cephalopods: 2.0	Fish: 0.5 carnivorous fish: 1.0 Tuna: 1.2 Alfonsino: 1.5 marlin: 1.7 Shark: 1.6	Fish: 0.1	aquatic animal: 2.0
Korea	General food standards and specifications(2014–11-04)	Fish: 0.5 Crustaceans: 1.0 Bivalve mollusks: 2.0	Fish: 0.1 crustaceans: 1.0 algae: 0.3 bivalve molluscs: 2.0	Fish: 1.0 bivalve molluscs: 0.5		
United States	United States Pharmacopoeia(USP 35-NF 30)	crustaceans: 1.5 bivalve molluscs: 1.7	crustaceans: 3.0 shellfish: 4.0	Fish: 1.0		

## Removal techniques of heavy metals and harmful elements in MTCMs

Effective control of heavy metals and harmful elements in MTCMs is crucial for the future development of MTCMs in China, as heavy metals and harmful elements can cause damage to the marine environment, marine organisms and human bodies to varying degrees, which seriously affects the safety and consumption of MTCMs. In recent years, the removal of heavy metals and harmful elements in traditional Chinese medicines has received significant attention from researchers, resulting in the development of several related heavy metal control technologies. Animal medicines constitute about 2/3 of MTCMs, and they are known to accumulate certain heavy metal elements in their bodies easily. Moreover, after metabolism, the speciation of elements such as As and Hg can change, leading to poisoning. Therefore, when studying the control technology of heavy metals and harmful elements in MTCMs, specific animals and specific elements should be analyzed differently to achieve effective removal of heavy metals.

The removal of heavy metals and harmful elements in MTCMs has become a key area of focus in recent years due to their detrimental effects on the marine environment, marine organisms, and human health. Traditional physical and chemical methods for removing heavy metals have not yielded significant results, such as chemical precipitation method and ion exchange method, leading to the development of more effective removal technologies. Currently, two main categories of removal methods are used: in vivo heavy metal purification and proteolysis heavy metal removal. In vivo methods include various approaches such as purified water temporary culture, aquaculture water purification, feed purification, and microbial preparation purification, etc. Proteolysis methods include adsorption, complexation, chelating resin, and membrane separation, etc., as shown in Table 3. Recent research has shown promising results for the removal of heavy metals: Lin et al. [77] used waste oyster shells to prepare vaterite calcium carbonate particles to remove heavy metal ions. The results demonstrated that this method had excellent performance for removing heavy metal ions: Pb^2+^ (99.9%), Cr^3+^(99.5%). Jing et al. [78] demonstrated that bioremediation is a reliable and feasible method for treating Cr (VI) in water bodies. However, this method has limitations such as poor tolerance. To address this issue, they proposed microbial immobilization technology as an efficient method for removing Cr (VI). Similarly, Pak et al. [79] demonstrated that nano-remediation can effectively remove heavy metal pollutants by adsorption and catalytic reactions, converting them to stable metal states. Manirethan et al. [80] used a biosynthetic melanin-coated PVDF membrane to remove Cr (VI), Pb (II), Hg (II) and Cu (II) from aqueous solutions. The results showed that melanin-coated PVDF membrane had excellent heavy metal ion removal performance, with removal rates of Hg, Cr, Pb and Cu being 87.6%, 88.45%, 91.8% and 95.8%, respectively. Chinnadurai et al. [81] investigated the effects of temperature, salinity and body size on heavy metal removal from India clams, mussels and ostreae concha by a static purification system. Their findings suggested that medium-sized bivalve marine animals are most effective in removing heavy metals at room temperature (30 ± 1 °C) and a salinity range of 25–35 psu.

**Table 3:** Common heavy metal removal methods

Methods	Category	Principle	Advantages	Disadvantages	References
In vivo heavy metal purification method	Water purification temporary method	Place contaminated seafood in a clean water area for incubation, and excrete heavy metals through the metabolism of the organism	Simple operation	Long time consuming, High loss, Easy to cause water pollution	[93] [94]
	Aquaculture water purification method	Keep the water clean to reduce pollution	Titanium nanomaterials have large specific surface area and thermal stability	Expensive	[95] [96] [97]
	Feed purification method	Adding Polysaccharide Complex Bait to Purified Water for Temporary Feeding, and excrete heavy metals through the metabolism of the organism	Improve removal efficiency	–	[98] [99]
	Microbial preparation purification method	Add microbial agents in the water body to balance the flora and inhibit the propagation of harmful bacteria	Reduce harmful substances and save resources	–	[100]
Removal of Heavy Metals by Protease Hydrolysis	Adsorption method	The heavy metals are adsorbed on the surface with adsorbent, and the purpose of separating the active molecules from the heavy metals is achieved by desorbing the heavy metals	Wide source, low cost, no secondary pollution, strong heavy metal adsorption capacity (Chitosan)	Poor adsorption selectivity, narrow application range of pH value, etc	[101] [102] [103] [104]
	Complex method	Contact heavy metals with complexing reagents to form heavy metal complexes, so as to remove heavy metals	Non toxic, wide application range of pH value, strong chelating ability (phytic acid) Good combination ability	Long processing time	[105] [106]
	Chelating resin method	Heavy metal ions coordinate with resins with active atoms to form stable chelates	Good adsorption effect, high selectivity, good stability and good separation effect	Complex regeneration process	[107] [108] [109]
	Membrane separation method	Selective permeability of ion exchange membrane, including ED and UF	The removal effect is good by combining various technologies	–	[110] [111]

## Progress in quality control of heavy metals and hazardous elements in MTCM

### Current status of quality standards for MTCMs

To accurately understand the current stage of China's research on heavy metals and harmful elements in MTCMs, the authors conducted a search of the 2020 edition of the Chinese Pharmacopoeia and related standards for Chinese herbal medicine. The 2020 edition of the Chinese Pharmacopoeia includes a total of 616 types of Chinese medicines, of which 11 are MTCMs, accounting for only 1.79% of the total enrolled. These MTCMs include 6 types of animal shell MTCM (ostreae concha, haliotidis concha, arcae concha, margaritifera concha, meretricis concha cyclinae concha and sepiae endoconcha), 2 types of animal whole class MTCMs (syngnathus, hippocampus), 1 types of animals stimulated to form secretions-margarita, 2 plant-based MTCMs (sargassum, laminariae thallus eckloniae thallus). The authors found that, among the 11 MTCMs included in the 2020 edition of the Chinese Pharmacopoeia, only 6 have established inspection items for heavy metals and harmful elements in terms of quality standards, namely meretricis concha cyclinae concha, sepiae endoconcha, sargassum, laminariae thallus eckloniae thallus, ostreae concha and margarita. The remaining 5 MTCMs do not have this inspection item, as shown in Table 4. From Table 4, it can be seen that all 11 MTCMs contain trait identification items. However, only syngnathus have trait identification items, hippocampus and arcae concha also have powder identification and microscopic identification, but lack of physicochemical identification and thin-layer identification, while sargassum only has powder identification and physicochemical identification, laminariae thallus eckloniae thallus contains only physicochemical identification, while sepiae endoconcha, meretricis concha cyclinae concha, ostreae concha, haliotidis concha, margarita and margaritifera concha all have powder identification, microscopic identification, and physicochemical identification. In addition, the alcohol-soluble leachate content is specified for sargassum and laminariae thallus eckloniae thallus. For sepiae endoconcha, meretricis concha cyclinae concha, ostreae concha, arcae concha, and haliotidis concha, the prescribed content determination index component is calcium carbonate. The content of sargassum is determined using fucose as an indicator component, and for laminariae thallus eckloniae thallus, there are two types of indicator components specified for content determination, namely iodine and fucose. The quality standards of heavy metals and harmful elements in MTCMs included in the Chinese Pharmacopoeia are shown in Table 5. Among the six MTCMs that have established limits for heavy metals and harmful elements, the maximum allowable Pb content in the body is 5.0 mg/kg. The maximum allowable Cd content varies according to species, with sepiae endoconcha not exceeding 5.0 mg/kg, sargassum and laminariae thallus eckloniae thallus not exceeding 4.0 mg/kg, and meretricis concha cyclinae concha, ostreae concha, and margarita not exceeding 0.3 mg/kg. These limits require special attention. The maximum allowable As content in cuttlebone is 10 mg/kg, while meretricis concha cyclinae concha, ostreae concha, and margar ita should not exceed 2.0 mg/kg, and the arsenic content of sargassum and laminariae thallus eckloniae thallus is not clearly specified. The maximum allowable Hg content in sepiae endoconcha, meretricis concha cyclinae concha, ostreae concha, and margarita is 0.2 mg/kg, while in sargassum and laminariae thallus eckloniae thallus, it is 0.1 mg/kg, which also requires attention. Finally, the maximum allowable Cu content in the body is 20 mg/kg.

**Table 4:** Quality standards of MTCM included in the 2020 edition of the Chinese Pharmacopoeia and related standards for Chinese herbal medicines

MTCMs (names)	Qualitative identification items	Inspection items	Leachate	Content determination
Syngnathus	Characteristic	Moisture	–	–
Hippocampus	Characteristic, Powder, Microscopic	Dry weight reduction, Total Ash, Acid-insoluble ash	–	–
Sepiae Endoconcha	Characteristic, Powder, Microscopic, Physicochemical	Heavy metals and harmful elements, Moisture, Dry weight reduction, Acid-insoluble ash	–	Calcium carbonate (CaCO_3_)
Sargassum	Characteristic, Powder, Physicochemical	Heavy metals and harmful elements, Moisture, Total Ash, Acid-insoluble ash	Alcohol-soluble leachate	Fucose (C_6_H_12_O_5_)
Meretricis Concha Cyclinae Concha	Characteristic, Powder, Microscopic, Physicochemical, Thin-layer	Heavy metals and harmful elements, Acid-insoluble ash	–	Calcium carbonate (CaCO_3_)
Laminariae Thallus Eckloniae Thallus	Characteristic, Physicochemical	Moisture, Total Ash, Heavy metals and harmful elements	Alcohol-soluble leachate	Iodine, Fucose (C_6_H_12_O_5_)
Ostreae Concha	Characteristic, Powder, Microscopic, Physicochemical, Thin-layer	Heavy metals and harmful elements, Acid-insoluble ash, Dry weight reduction, Arsenic salt	–	Calcium carbonate (CaCO_3_)
Haliotidis Concha	Characteristic, Powder, Microscopic, Physicochemical	Dry weight reduction, Acid-insoluble ash	–	Calcium carbonate (CaCO_3_)
Arcae Concha	Characteristic, Powder, Microscopic			Calcium carbonate (CaCO_3_)
Margarita	Characteristic, Powder, Microscopic, Physicochemical	Heavy metals and harmful elements, Acid-insoluble ash		
Margaritifera Concha	Characteristic, Powder, Microscopic, Physicochemical	Acid-insoluble ash

**Table 5:** Quality standards of heavy metals and harmful elements in marine Chinese medicine in the 2020 edition of the Chinese Pharmacopoeia

MTCMs (names)	Inspection items: heavy metals and harmful elements	Lead(mg/kg)	Cadmium(mg/kg)	Arsenic(mg/kg)	Mercury(mg/kg)	Copper(mg/kg)
Syngnathus	-	–	–	–	–	–
Hippocampus	-	–	–	–	–	–
Sepiae Endoconcha	√	5	5	10	0.2	20
Sargassum	√	5	4	-	0.1	20
Meretricis Concha Cyclinae Concha	√	5	0.3	2	0.2	20
Laminariae Thallus Eckloniae Thallus	√	5	4	-	0.1	20
Ostreae Concha	√	5	0.3	2	0.2	20
Haliotidis Concha	-	–	–	–	–	–
Arcae Concha	-	–	–	–	–	–
Margarita	√	5	0.3	2	0.2	20
Margaritifera Concha	–	–	–	–	–	–

### On the current problems in the quality standards of MTCMs

After conducting a search of the 2020 edition of the Chinese Pharmacopoeia, the standards for Chinese herbal medicines in coastal areas, and the specifications for the preparation of Chinese medicinal tablets, the author discovered that the number of MTCMs included in coastal provinces was higher than those included in the Chinese Pharmacopoeia. However, it still differs greatly from the number of MTCMs recorded in the contemporary marine work “Chinese Marine Materia Medica”. Therefore, there are several issues with the current quality standards of MTCMs as follows: Firstly, the number of MTCMs included in the Chinese Pharmacopoeia and related Chinese herbal medicine standards is insufficient, and the quality standards of the included MTCMs are severely lacking. This significantly impacts the clinical application and long-term development of MTCMs. Secondly, using a single component as the detection index for quality control of TCM does not align with the characteristics of TCM, which have multiple targets and efficacy, and fails to comprehensively represent the intrinsic quality and clinical efficacy of TCM. Lastly, metal elements play a vital role in evaluating the efficacy and safety of MTCMs. On the one hand, the content distribution of metal elements in MTCMs has a crucial influence on its clinical efficacy, and the speciation changes of metal elements will affect the efficacy of the elements. On the other hand, metal elements significantly impact the safety evaluation of MTCMs.

## Establish a pollution detection database and quality and safety supervision system for MTCMs

### Establishing a pollution detection database for MTCMs

Although there has been an increase in domestic and foreign research on the marine environment and detection technology for heavy metals in MTCM, certain results have been achieved. However, with the recent and the increasingly serious pollution of the marine environment, leading to a rise in MTCM pollution levels, it is imperative to conduct more targeted research on the control technology of heavy metals and harmful elements in different MTCM, and conduct a more realistic assessment of the health risk associated with MTCM. Establishing a pollution detection database of MTCM is necessary to more accurately understand the pollution status of MTCM in different regions. On the one hand, this can promote the development of MTCM, break the bottleneck of the development of MTCM in China, and provide guidance for the formulation of limits on heavy metals and harmful elements in MTCM. On the other hand, it can also provide a reference for the rational use of MTCM in people's medicine, ensuring its the safety and effectiveness.

### Norm for processing of MTCMs

During the production and processing of Chinese medicinal materials, it is crucial to prevent the introduction of heavy metal debris resulting from improper processing such as cutting and crushing. Additionally, it is important to thoroughly inspect the excipients used in production to ensure that they are free of any heavy metal residue. Before packaging the final product, it is necessary to strictly check that all materials meet the standards outlined in the Technical Specification for Packaging of Chinese Medicinal Materials, and that the production process follows the flow specified in GMP. When storing and transporting the medicinal materials, it is important to keep them dry and in a vacuum. Additives such as coal and sulfur should be used appropriately, and unreasonable drying methods should be avoided to prevent excessive heavy metal pollution, such as As, Cd, and Pb, from being introduced.

### Conditions for storage of MTCMs

When storing Chinese medicinal materials, it is essential to ensure the cleanliness of the storage area to prevent heavy metal pollution caused by improper storage. Scientific and reasonable drying methods should be employed, while avoiding or minimizing the use of unreasonable drying methods. Storage conditions must be strictly implemented according to the Technical Specification for Chinese Medicinal Materials Warehouse and the Standard for Storage Management of Chinese Medicinal Materials. Additionally, technical training for supervisors should be strengthened to prevent pollution caused by human factors. This training will not only help to prevent pollution caused by human error but also to enable supervisors to deal with any emergencies that may arise, thus strengthening the modern scientific management.

### Establishment of comprehensive quality and safety supervision system of MTCMs

Currently, although there have been some achievements in the detection and analysis of heavy metals and harmful elements in MTCMs, it is still far from enough. Many detection technologies used in MTCMs are copied from other industries, such as food and water quality detection, and few detection technologies are tailored to the characteristics of MTCMs. Moreover, the risk assessment of MTCMs is inadequate, which has hindered the development of MTCMs. Therefore, establishing a safe and feasible quality and safety supervision system of MTCM with a trinity of “production specification-testing platform-risk assessment” is of great importance. For MTCMs, quality and safety depend on standardized production and a healthy marine ecological environment. Testing technology is a critical means of identifying the quality and safety of traditional Chinese medicine, and assessing the targeted risks is necessary to determine whether the quality of traditional Chinese medicine is safe or not. Only by establishing a comprehensive quality and safety supervision system that is truly tailored to MTCMs can we maximize the quality and safety of MTCMs.

## Conclusions

This review discusses the characteristics of heavy metals in MTCMs, including their current research status, hazards, detection techniques, removal techniques, speciation, risk assessment and limit standards. Considering the current research status in MTCMs, the authors propose a series of research directions for heavy metals and harmful elements in MTCMs, with the aim of providing references for the ongoing development and application of MTCMs: (1) Establish corresponding elemental fingerprint maps for MTCMs in different oceanic regions and geographic locations, study the distribution of elements in MTCMs to clarify the relationship between element content, distribution, and geographical location in MTCMs. Provide basis for quality determination of MTCMs and provide technical means and reference for authenticity identification of MTCMs. (2) In MTCMs, heavy metal elements often exist in multiple speciations, with varying toxicity and stability. The harm to human health also varies accordingly. Therefore, research on element analysis and speciation of heavy metals (such as As, Hg, etc.) in MTCMs should be intensified, aiming to scientifically assess the risks of heavy metals and harmful elements in MTCMs, clarify their degree of harm to human health, and promote the development and clinical application of MTCMs. (3) Currently, the detection and removal technologies for heavy metals and harmful elements in MTCMs are still not perfect. Therefore, while drawing on relevant heavy metal detection technologies from traditional Chinese medicine, food, and agriculture industries, it should be recognized that MTCMs have their own characteristics. Therefore, research and development of heavy metal detection and removal technologies that are in line with the characteristics of MTCMs should be conducted to ensure the rapid development of MTCMs. (4) Pollution assessment should be conducted for marine medicinal herbs from different oceanic regions and different species to clarify the ecological risk index and pollution level of MTCM in the study sea area, providing reference for the government to formulate corresponding decisions and plans. (5) Due to the large number of MTCMs and the complex types of heavy metals, the scientific assessment of the health risks of heavy metals and harmful elements in MTCMs is of great significance. Therefore, a MTCMs quality and safety supervision system that integrates "production standards—detection platform—risk assessment" should be established, comprehensively monitoring the quality and safety of MTCMs from the aspects of production, detection, and risk assessment, in order to ensure the quality and safety of MTCMs.

## Data Availability

All data are fully available without restriction.

## Abbreviations

MTCM: Marine traditional Chinese medicine
TCM: Traditional Chinese medicine
TAM: Traditional animal medicines
ICP-MS: Inductively coupled plasma mass spectrometry
THQ: Target hazard quotient
EDI: The estimated daily intake
CR: Cancer risk
